# Upregulation of Long Non-Coding RNA DRAIC Correlates with Adverse Features of Breast Cancer

**DOI:** 10.3390/ncrna4040039

**Published:** 2018-12-11

**Authors:** Dan Zhao, Jin-Tang Dong

**Affiliations:** 1 Department of Genetics and Cell Biology, College of Life Sciences, Nankai University, Tianjin 300071, China; jtdong@nankai.edu.cn or j.dong@emory.edu; 2 Department of Hematology and Medical Oncology, School of Medicine, Winship Cancer Institute, Emory University, Atlanta, GA 30322, USA

**Keywords:** lncRNA, *DRAIC*, *LOC145837*, *RP11-279F6.1*, breast cancer

## Abstract

*DRAIC* (also known as *LOC145837* and *RP11-279F6.1*), is a long non-coding RNA associated with several types of cancer including prostate cancer, lung cancer, and breast cancer. Its expression is elevated in tumor tissues compared to adjacent benign tissues in breast cancer patients and is regulated by estrogen treatment in breast cancer cells. In addition, expression analysis of *DRAIC* in more than 100 cell lines showed that *DRAIC* expression is high in luminal and basal subtypes compared to claudin low subtype, suggesting a prognostic value of *DRAIC* expression in breast cancer. In the present study, we analyzed *DRAIC* expression in 828 invasive breast carcinomas and 105 normal samples of RNA sequencing datasets from The Cancer Genome Atlas (TCGA) and found that *DRAIC* expression was correlated with estrogen receptor (*ER*), progesterone receptor (*PR*), and human epidermal growth factor receptor 2 (*HER2*) status, and is increased in cancerous tissues. Additionally, higher *DRAIC* expression was associated with poorer survival of patients, especially in ER positive breast cancer. *DRAIC* was also investigated in the Oncomine database and we found that *DRAIC* expression predicted patients’ response to paclitaxel and FEC as well as lapatinib, which are commonly used therapy options for breast cancer. Finally, *DRAIC* expression in breast cancer was negatively correlated with immune cell infiltration. These results reinforce the importance of *DRAIC* in breast cancer.

## 1. Introduction

Over the past few years, there has been an increasing widespread interest in long non-coding RNAs (lncRNAs), which are non-protein coding transcripts more than 200 nucleotides in length [1,2]. They are now known as important players in human diseases including various types of cancers [3,4,5,6]. They were reported to mediate important cellular functions like genomic imprinting, X-chromosome inactivation, and RNA splicing. They modulate gene expression at the epigenetic, transcriptional, and post-transcriptional levels through different mechanisms like enhancer-associating RNAs, chromatin looping, transcription factor trapping, and acting as competing endogenous RNA (ceRNA, a.k.a. RNA sponges) interacting and sequestering microRNAs (miRNAs) [7,8].

*DRAIC* (a.k.a. *LOC145837* and *RP11-279F6.1*) is a 1.7 kb long noncoding RNA. It, together with another noncoding RNA gene *PCAT29*, locates at 15q23. This locus was first found to be a tumor suppressor in prostate cancer [9]. In breast cancer, Lee reported that *DRAIC* was a potential oncogene and using more than 100 breast cancer cell lines they showed that *DRAIC* was high in luminal and basal subtypes compared to the claudin low subtype [10]. They carried out in vitro studies and showed that knockdown *DRAIC* significantly inhibited cell proliferation by regulating cell cycle and E2-dependent signaling. They also confirmed their finding in patients’ samples and included 135 tumor tissues and 27 benign tissues, showing *DRAIC* mRNA expression is indeed higher in tumor tissues [10]. However, they failed to examine *DRAIC* expression in different subtypes and correlate it to clinically prognostic and pathological parameters, possibly due to the small number of samples analyzed.

In the present study, we investigated *DRAIC* expression in 828 invasive breast carcinoma samples and 105 normal samples from The Cancer Genome Atlas (TCGA) and confirmed *DRAIC* expression was higher in tumor tissues than normal tissues. We also described that *DRAIC* expression is significantly higher in estrogen receptor (ER) positive, progesterone receptor (PR) positive, and human epidermal growth factor receptor 2 (HER2) positive patients compared to their negative counterparts. *DRAIC* expression correlated with tumor stage and lymph node metastasis. Higher expression of *DRAIC* also predicts poorer overall survival and disease specific survival of breast cancer patients especially in ER positive subtypes. Additionally, we investigated *DRAIC* expression in Oncomine database and found *DRAIC* expression also predicts patients’ response to anticancer drugs like paclitaxel and FEC (Fluorouracil, Epirubicin, and Cyclophosphamide, which are commonly used drugs in chemotherapy) and lapatinib (which is a used to treat women with HER2 positive breast cancer). Finally, *DRAIC* is investigated in TIMER and interestingly its expression predicts immune cell infiltration levels in breast cancer. These results indicate *DRAIC* plays an important role in breast cancer and may be a potential therapeutic target or a prognostic marker.

## 2. Results

### 2.1. DRAIC Expression Increased in Breast Cancer and Correlated with Estrogen Receptor, Progesterone Receptor, and Human Epidermal Growth Factor 2 and Tumor Stages and Lymph Node Metastasis

**Figure 1:**
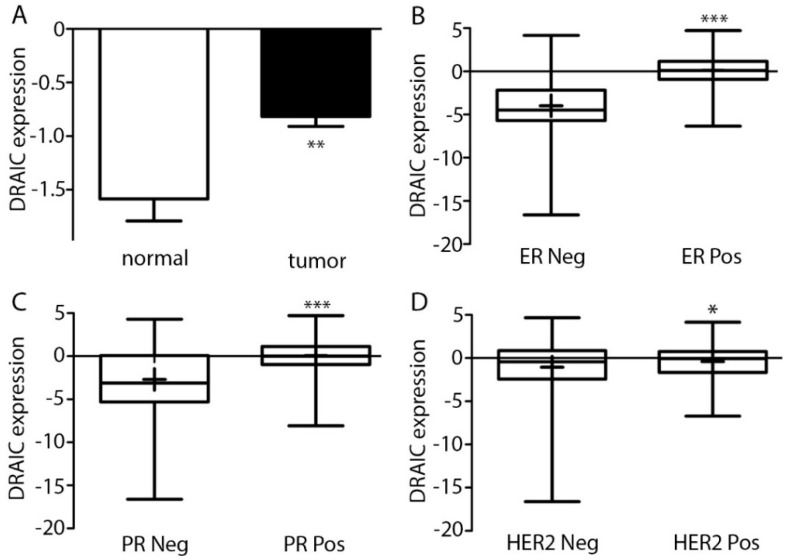
*DRAIC* expression is high in breast cancer compared with normal tissues and its expression correlates with important breast cancer marker genes estrogen receptor (*ER)*, progesterone receptor (*PR)*, and human epidermal growth factor receptor (*HER2)*. (**A**) *DRAIC* expression was analyzed in 828 breast cancer patients and 105 normal breast tissue samples, the data shown are mean ± SEM (standard error of the mean). (**B**–**D**) The breast cancer patients were divided according to their ER, PR, and HER2 status, respectively. Shown is the Whisker plots: Min to max and for each group, the mean value was shown as “+”. Statistical significance was calculated by two-tailed student’s *t*-test, * indicates *p* < 0.05, ** indicates *p* < 0.01, and *** indicates *p* < 0.001. *DRAIC* expression was shown as log2 value.

**Figure 2:**
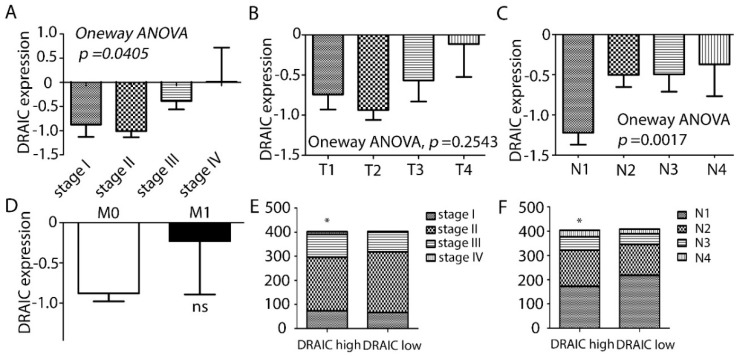
High *DRAIC* expression in breast cancer correlates with certain clinical pathological parameters. (**A**–**D**) The 828 breast cancer patients were divided according to their neoplasm disease stages (**A**), cancer tumor stages (**B**), neoplasm disease lymph node stages (**C**), and cancer metastasis stages (**D**). *DRAIC* expression in each different group of stages was shown as mean ± SEM (standard error of the mean). Statistical significance was calculated by either one-way ANOVA (for **A**–**C**) or two-tailed student’s *t*-test (for **D**). (**E**,**F**) The 828 breast cancer patients were divided into *DRAIC* high and *DRAIC* low group based on the median value of *DRAIC* expression. In each group, a stacked bar chart was created to show the distribution of patients in different neoplasm disease stages (**E**) or lymph node stages (**F**). Chi-square tests were used to test the differences. * indicates *p* < 0.05; ns, not significant. *DRAIC* expression was shown as log2 value.

The expression of *DRAIC* was investigated in 828 tumor tissues and 105 normal breast tissues. We showed that *DRAIC* expression was significantly higher in cancer tissues than normal tissues (Figure 1A), consistent with a previous report [10]. Breast cancer is closely related to hormone signaling and EGF signaling and could be divided into several subtypes based on the expression of ER, PR, and HER2 [11]. We divided the 828 samples according to their ER, PR, and HER2 status and expression of *DRAIC* in these subgroups showed that *DRAIC* expression is higher in ER, PR, and HER2 positive patients compared to their negative counterparts (Figure 1B–D). We found *DRAIC* expression was higher in late stage tumors compared to early stages and higher expression of *DRAIC* is detected in lymph node positive patients compared to lymph node negative patients, indicating *DRAIC* may be involved also in the metastatic progression of breast cancer. When only tumor size (the T stage) or long-distance metastasis (M stage) were considered, a trend was also found that higher *DRAIC* indeed was bad for the patients (Figure 2A–D). We divided the 828 patients into *DRAIC* high and *DRAIC* low and we found that in *DRAIC* high group, there is a significant higher proportion of late stage and lymph node metastasis patients (Figure 2E,F).

### 2.2. DRAIC Expression Impact Patients Survival

**Figure 3:**
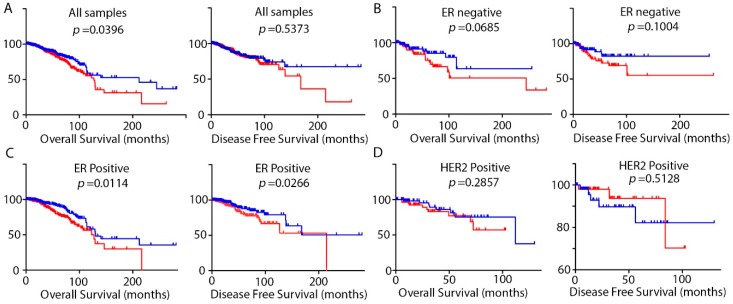
Overall survival or disease-free survival determined by Kaplan–Meier plots and the log-rank test according to *DRAIC* expression. The patients were divided as *DRAIC* high (red) or *DRAIC* low (blue) according to the median value of *DRAIC* expression. Kaplan–Meier plots were created using GraphPad Prism 5 software using data for all samples (**A**) or only ER negative patients (**B**), ER positive patients (**C**), and HER2 positive patients (**D**). Log-rank tests were used to calculate the differences between groups.

Then we investigated the impact of the expression of *DRAIC* on the overall survival and disease specific survival of the patients. We found that in general, higher *DRAIC* predicts shorter overall survival of patients (Figure 3A). Detailed analysis showed that higher *DRAIC* significantly predicts shorter overall survival and disease specific survival in ER positive patients but not in ER negative patients (Figure 3B,C), which is consistent with our finding that *DRAIC* is higher in ER positive patients than ER negative patients (Figure 1B). We also found that higher *DRAIC* in HER2 positive patients, in the contrary, showed better disease-free survival although no statistical significance was reached, suggesting a subtype specific impact (Figure 3D).

### 2.3. DRAIC Expression Predicts Patients’ Response to Chemotherapy Treatments

**Figure 4:**
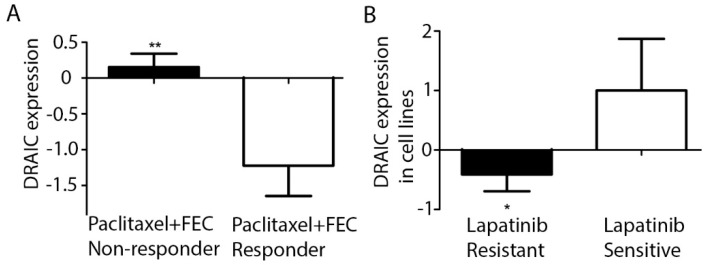
*DRAIC* expression correlates to chemotherapy treatment response. (**A**) *DRAIC* expression in 88 paclitaxel and FEC (fluorouracil/epirubicin/cyclophosphamide) non-responder patients and 27 paclitaxel and FEC responder patients’ samples (Miyake 2012) were shown as mean ± SEM. (**B**) *DRAIC* expression in 17 lapatinib resistant cell lines and 5 sensitive cell lines (Barretina 2012) were shown as mean ± SEM. Statistical significance was calculated by two-tailed student’s *t*-test, * indicates *p* < 0.05 and ** indicates *p* < 0.01.

We investigated *DRAIC* and its expression in the Oncomine database [12]. Interestingly, we found that *DRAIC* expression is higher in patients who are resistant to paclitaxel and FEC (F, Fluorouracil; E, Epirubicin Hydrochloride; C, Cyclophosphamide) (Figure 4A) [13]. All these drugs are commonly used chemotherapy drugs for treatment of breast cancer. We also found one report regarding breast cancer cell lines showing that *DRAIC* expression is higher in lapatinib sensitive breast cancer cell lines compared to Lap resistant cell lines (Figure 4B) [14]. Lapatinib is a commonly used anti-HER2 therapy drug in HER2 overexpressing breast cancer patients. These results indicate that *DRAIC* expression may help guide the treatment options for breast cancer patients. Its higher expression may render patients resistant to certain chemotherapy treatments while sensitize patients to other drugs.

## 3. Discussion

The lncRNA *DRAIC* was first identified in prostate cancer cell lines and showed higher expression in human LNCaP cells (which is androgen-dependent) compared with LNCaP-derived C4-2B cells (which is androgen independent). It was located in the 15q23 region, together with another lncRNA *PCAT29* [9]. Further, they discovered that *DRAIC* was downregulated by androgen and found androgen receptor (AR) and FOXA1 and NKX3-1 directly bound *DRAIC* promoters. AR inhibits *DRAIC* expression and FOXA1 and NKX3-1 upregulated *DRAIC* expression. Functional assays through overexpression or knockdown studies showed that *DRAIC* promoted proliferation but inhibited migration and invasion of LNCaP cells while *PCAT29* inhibited LNCaP proliferation. In a more recent paper, the authors have shown that a specific metabolite named 5-alpha-Abi of Abiraterone, which is a steroidgenesis enzyme inhibitor for the treatment of metastatic castration resistant prostate cancer, could induce *DRAIC* expression, indicating *DRAIC*’s involvement in anti-androgen therapy resistance for prostate cancer [15]. *DRAIC* expression was repressed by androgen treatment and antiandrogen treatment induced *DRAIC* expression [16]. It was also found *DRAIC* expression was upregulated in IR-resistant PCs through expression analyses of human prostate cancer xenografts with predetermined radioresistant/sensitive phenotypes [17].

Aberrant expression of *DRAIC* was also related to other types of cancers. For example, in one multiple type cancer study, the authors obtained 132 samples of paired tumor and normal adjacent tissues for four types of cancers (bladder, prostate, lung, and ER positive breast cancer), analyses of the RNA-seq data revealed *DRAIC* to be significantly upregulated in three of the four cancer types except bladder cancer [18]. In another study, the authors focused on gastrointestinal cancers (pancreas, liver, stomach, esophagus, and colorectal cancers), and used data from Oncomine database to identify genes that are dysregulated, among which DRAIC was discovered as one of the 28 downregulated genes unique to the pancreas cancers [19]. In colorectal cancers, *DRAIC* expression was higher in chemotherapy responding versus non-responding patients [20]. In lung cancer, *DRAIC* was identified as one of the 71 lncRNAs in non-small-cell lung carcinomas that could distinguish squamous cell carcinomas from adenocarcinomas [21] and was found to be one of the most significantly (top 5) downregulated lncRNAs in squamous lung cancer [22].

**Figure 5:**
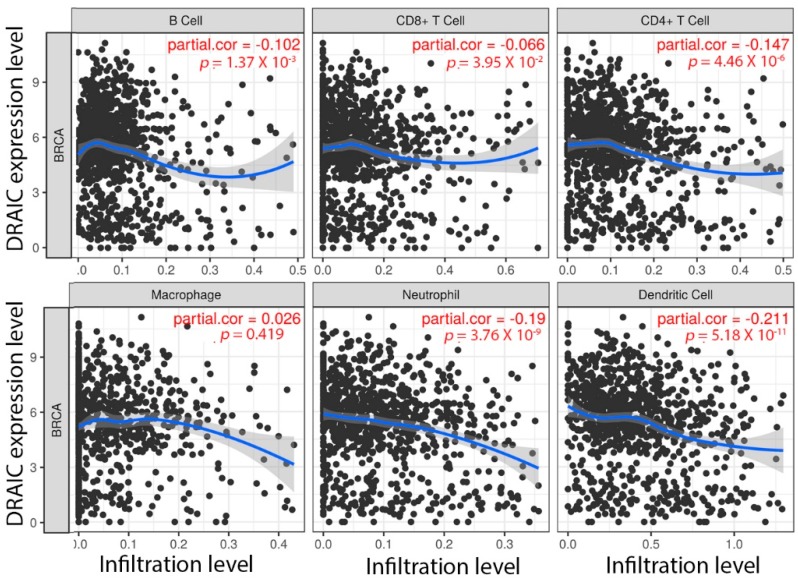
*DRAIC* expression negatively correlates with immune cell infiltration levels in breast cancer. The correlation between *DRAIC* expression and abundance of immune infiltrates is investigated through TIMER [27]. Correlation between *DRAIC* expression and the abundances of six immune infiltrates (B cells, CD4+ T cells, CD8+ T cells, Neutrophils, Macrophages, and Dendritic cells) are displayed. The purity-corrected partial Spearman correlation and statistical significance are shown on the top right corners.

In breast cancer, *DRAIC* expression was identified as a potential onco-lncRNA based on its elevated expression in tumor tissues compared to the adjacent normal tissues [23]. It was shown to be higher in ER positive breast cancer patients from 4 different studies [23,24,25,26]. However, while *DRAIC* knockdown in MCF7 cells inhibited E2-regulated gene expression, the expression of *DRAIC* itself was also inhibited by E2 treatment and it was speculated that DRAIC may be important for both E2-dependent and the basal growth of cancer cells [10]. By analyzing ChIP-seq data of ER and 26 known transcription factors, it was shown that there is direct ER binding on the genomic region of *DRAIC* and *DRAIC* expression also could be regulated by key transcription factors like GATA3, E2F1, MYC, and RAD21, which are key regulators in breast tumorigenesis [23]. *DRAIC* was annotated to be one of the 38 lncRNAs associated with ER and based on the “guilt-by-association” analyses, researchers revealed *DRAIC* to be potentially involved in inhibition of immunity, indicating *DRAIC* to be involved in both ER signaling and other processes beyond ER [25]. We explored *DRAIC* in TIMER [27], a web server developed by Harvard to explore the correlation between its expression and abundance of immune infiltrates. We found that higher *DRAIC* expression in breast cancer significantly negatively correlates with immune cell infiltration especially dendritic cells and neutrophils (Figure 5). Further studies are needed to investigate whether or not *DRAIC* really participates in immune responses of breast cancer patients and if it acts through cooperation with ER signaling (or HER2 signaling) or not. In another study, researchers constructed lncRNA-miRNA-mRNA ceRNA networks based on data obtained from 116 ER positive breast cancer samples and proposed that *DRAIC* may function as miRNA sponge and exert its effects through mir-296-3p, mir-432-5p, and mir-149-5p [24].

In this study, we included expression analysis of *DRAIC* in 828 tumor samples and 105 normal breast samples from TCGA. This large number of samples enables broad analyses and allowed us to analyze *DRAIC* expression in more detail, for example, in only ER positive patients.

High expression of *DRAIC* in breast cancer tissues compared to normal breast tissues was validated in the present study, confirming its role as a potential onco-lncRNA in breast cancer (Figure 1A). The present study, which was based on TCGA analysis, also described high *DRAIC* expression in ER positive, PR positive, and HER2 positive patients compared to their receptor negative counterparts (Figure 1). Detailed analyses revealed high *DRAIC* expression to be correlated to tumor stages and lymph node metastasis of the patients (Figure 2). Survival analysis considering all patients with breast cancer revealed high expression of *DRAIC* was significantly associated with poor overall survival. Detailed analyses revealed that high *DRAIC* expression was associated with poor survival only in ER positive patients but not in ER negative or HER2 positive patients (Figure 3), which could be related to its involvement in estrogen and ER regulated gene expression as reported previously [10]. Very interestingly, in HER2 positive patients, 5-year DFS is better in *DRAIC* high patients (93.7% in *DRAIC* high group compared to 82.2% in *DRAIC* low group, Figure 3D). Although no significance was reached, which could be due to smaller number (only 129) of HER2 positive patients in this study, this may indicate a subtype specific effect of *DRAIC* in breast cancer and its function in HER2 positive patients need to be investigated further.

*DRAIC* was also investigated in the Oncomine database and very intriguingly, we found its high expression may indicate resistance to paclitaxel and FEC while on the contrary, sensitivity to lapatinib, which inhibits HER2 and is widely used as anti-HER2 therapy in HER2 overexpressing breast cancer patients. This may partially explain why its high expression in HER2 positive patients showed better (although not significant) disease-free survival. In another study [24], researchers have found *DRAIC* to be lower in HER2 positive (which is contrary to our results) and higher in ER positive (which is similar to our findings) patients’ samples, but *DRAIC* didn’t show an effect on survival in their researches. This could be due to much smaller cohort size of their study compared to ours (148 vs 828), especially in HER2 positive patients (38 vs 129). Further studies with larger patients number especially in HER2 positive samples are needed to address *DRAIC* function in HER2+ breast cancer.

In conclusion, *DRAIC* expression was found to be higher in breast cancer patients, especially in ER, PR, and HER2 positive patients. Its expression correlates with tumor stages and lymph node metastases and patients’ survival especially in ER positive patients. *DRAIC* expression may also predict responsiveness to chemotherapy and anti-HER2 therapy and immune therapy. Additional studies are needed to better understand how *DRAIC* is involved in breast cancer and what molecular pathways are involved.

## 4. Materials and Methods

*DRAIC* expression for 828 invasive breast carcinomas and 105 normal breast tissues was collected by the open-access web resource TANRIC [28], which assessed and analyzed the breast cancer TCGA data. Clinical data for the patients were obtained from the open access cBioportal database [29,30]. Statistical analyses were carried out similar to a previous paper [31], briefly, two-tailed student’s t-test was used to assess statistical differences between two groups (for example, negative and positive ER, PR, HER2 status). One-way ANOVA followed by Bonferroni test was used to assess statistical differences for more than three groups.

Survival analyses were carried out similar to a previous paper [32], patients were divided into two groups (one above the median and the other below). The Kaplan–Meier curves and log-rank tests were used to estimate overall and disease-free survival of the patients.

Datamining in Oncomine database was performed by using *DRAIC* or *LOC145837* or *RP11-279F6* as keywords and related breast cancer studies were chosen for further look in details.

Data were analyzed using the GraphPad Prism 5 software and was considered to be significantly different when *p* was less than 0.05.
